# Diffusion models learn underlying trends in actomyosin networks and predict behavior at unseen filament turnover

**DOI:** 10.64898/2026.05.26.727950

**Published:** 2026-07-27

**Authors:** Elisabeth Rennert, Agnish Kumar Behera, Yuqing Qiu, Suriyanarayanan Vaikuntanathan

## Abstract

Generative diffusion models have demonstrated an ability to produce novel images sampled from the learned underlying data distribution. These models are able to infer system characteristics for parameter combinations that were not seen during training. We investigate the ability of these models to infer trends in biological data from limited samples. Specifically, we consider the response of system scale behaviors such as cortical flow in a simulated actomyosin system as we tune filament turnover rates. We train a diffusion model on coarse grained actin curvature and density heatmap images, and are able to generate images from conditioning variables not seen during training. These images are predictive of nonlinear trends in the system. We also consider characteristics of the system that allows this level of inference, such as the strong linear relationship between average density and filament turnover in the system, and explore minimal underlying dynamics with a motor binding model.

## Full Text Availability

The license terms selected by the author(s) for this preprint version do not permit archiving in PMC. The full text is available from the preprint server.

